# A Rare Case of Extensive Cerebral Venous Sinus Thrombosis Complicated by Heparin-Induced Thrombocytopenia

**DOI:** 10.1155/2022/7845786

**Published:** 2022-06-06

**Authors:** Noman Ahmed Jang Khan, Ashar Farooqi, Mohamed Alsharedi

**Affiliations:** Joan C. Edwards School of Medicine at Marshall University, Huntington, WV 25701, USA

## Abstract

Cerebral venous sinus thrombosis (CVST) is a rare but potentially life-threatening cause of stroke. Several risk factors have been identified including hypercoagulable state, malignancy, use of oral contraceptives, pregnancy, head injury, infection, and prothrombotic states such as heparin-induced thrombocytopenia (HIT). HIT is a prothrombotic state leading to thrombosis in several distinct locations including CVST requiring prompt discontinuation of heparin and initiation of nonheparin anticoagulation to prevent catastrophic consequences. Very rarely, HIT can complicate the ongoing CVST leading to worsening thrombosis and clinical deterioration. We here report an exceedingly rare case of CVST complicated by HIT in a 22-year-old female patient who showed remarkable clinical improvement after discontinuation of heparin and initiation of argatroban.

## 1. Introduction

Cerebral venous sinus thrombosis (CVST) is a rare but life-threatening cause of cerebrovascular events [1]. Anticoagulation remains the mainstay treatment for CVST even in cases with associated intracerebral hemorrhage [2]. Heparin is one of the most used anticoagulation agents used in the treatment of CSVT. In rare instances, heparin can cause heparin-induced thrombocytopenia (HIT) which can lead to worsening of ongoing thrombosis. We here report a case of a genetically predisposed patient who developed extensive CVST due to use of vaginal estradiol ring, in whom heparin treatment was complicated by HIT. Very few similar cases have been reported in the literature [3]. 

## 2. Case Presentation

**Figure 1:**
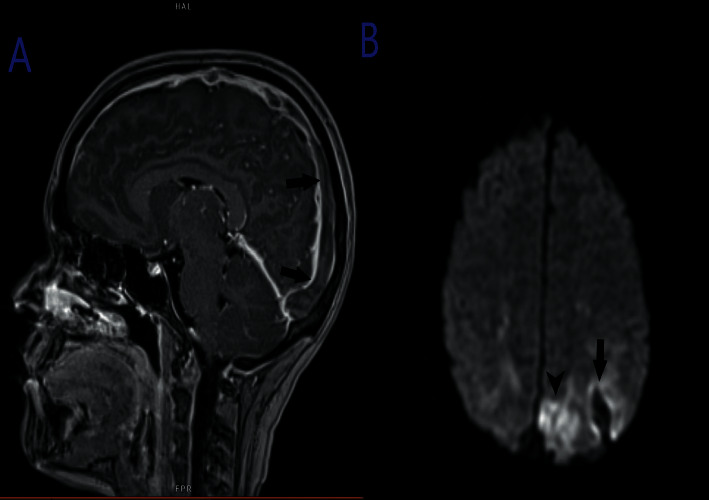
Magnetic resonance imaging (MRI) of the brain with contrast. (a) Postcontrast sagittal view showing extensive filling defect in the superior sagittal sinus consistent with thrombosis. (b) Diffusion weighted imaging (DWI) coronal view demonstrating left parietal lobe infarct (arrow) and hemorrhage (arrowhead).

**Figure 2:**
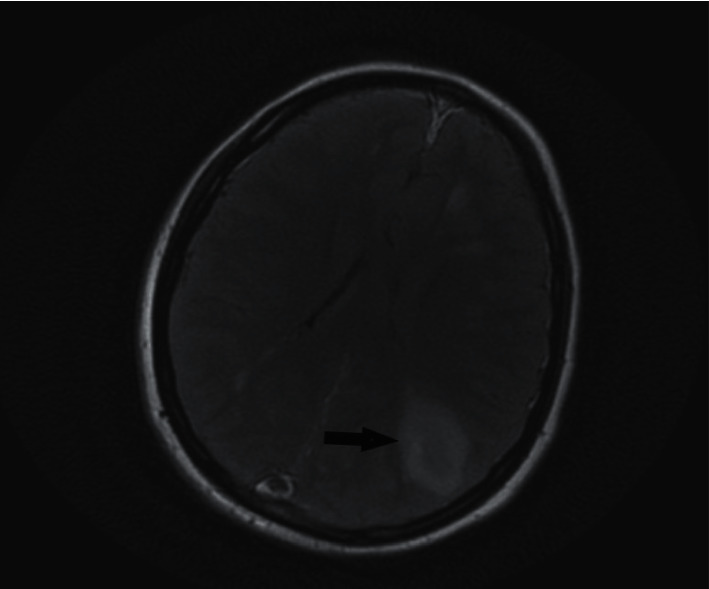
Magnetic resonance imaging (MRI) of the brain with contrast, T2 flair showing left parietal lobe hemorrhagic infarct (arrow).

**Figure 3:**
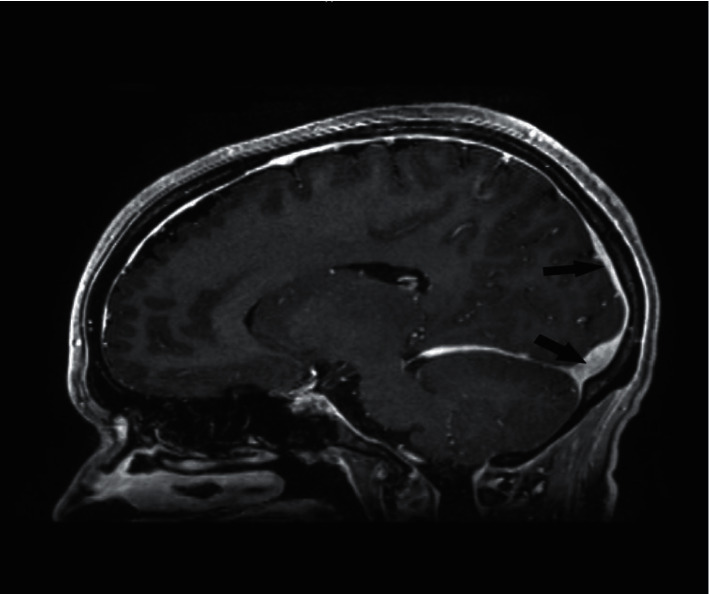
Magnetic resonance imaging (MRI) of the brain with contrast, postcontrast sagittal view showing marked improvement in the previously seen filling defect in the superior sagittal sinus (arrows).

A 22-year-old right-handed female was brought to the hospital with generalized tonic clonic seizures and reduced level of consciousness. Her initial vitals were temperature 98 F, blood pressure 110/67, pulse 105 bpm, and respiratory rate 20 breaths/min. Initial laboratory workup including complete blood count, electrolytes, creatinine, and liver function panel were unremarkable. During the initial neurological exam, the patient was stuporous and minimally responsive to verbal and tactile stimulation. On motor and sensory testing, she grimaced and appropriately withdrew to noxious stimuli in all four extremities. No nuchal rigidity or other signs of meningism were noted. No prior history of central nervous system abnormalities was reported. The patient had an etonogestrel/ethinyl estradiol vaginal ring for the past few months, and family history was also significant for unprovoked deep vein thrombosis (DVT) at an early age in a maternal uncle. Initial magnetic resonance imaging (MRI) brain with and without contrast demonstrated extensive thrombosis and complete occlusion of the superior, straight, left transverse, and sigmoid sinuses extending into the left cortical draining veins proximally and into the left internal jugular vein distally (Figure 1). FLAIR sequence also showed cortical edema in left posterior parietal region correlated with an area of diffusion restriction on DWI sequence which raised concern for venous infarction (Figure 2). Due to the extensive CVST, the patient was started on unfractionated heparin therapy with frequent/interval monitoring of activated partial thromboplastin time (aPTT) and the vaginal ring was removed. Mannitol was started for persistently elevated intracranial pressure (ICP). Video electroencephalogram (vEEG) findings were suggestive of encephalopathy with a potential epileptic focus in the left temporal region. No clinical electrographic seizure was noted, and the patient was kept on levetiracetam for seizure prophylaxis. Subsequently, hypercoagulability workup showed positive Factor V Leiden heterozygous mutation. Despite treatment with anticoagulation (AC) and hyperosmolar therapies, no improvement was appreciated in the patient's overall condition. Cerebral angiography with endovascular thrombolysis was attempted twice to recanalize but were not successful in relieving the clot burden. After 6 days of continuous heparin administration, there was a sudden drop in platelet counts, i.e., from 217,000 to 117,000 per microliter of blood. The 4T score was 5, indicative of intermediate probability HIT. Heparin was immediately stopped for a presumptive diagnosis of HIT. Repeat MRI brain with and without contrast showed persistent thrombosis and infarct (Figure 2). Due to extensive thrombosis and ongoing need for AC, the patient was started on parenteral argatroban and a HIT panel was sent. The platelet factor 4 (PF-4) assay came back elevated at 1.211 OD (optical density) which was later confirmed with a strongly positive serotonin release assay (SRA). Patient's clinical condition markedly improved and became more awake and responsive in the next few days. Repeat MRI brain demonstrated improvement in clot burden and markedly reduced density of thrombosis with much improved intraluminal contrast flow (Figure 3). Her platelets rebounded on argatroban, and a decision was made to switch to direct oral AC such as apixiban. She was eventually discharged in a stable condition.

## 3. Discussion

CVST is a life-threatening venous thrombosis which accounts for approximately 0.5 to 1% of all strokes [1]. Several risk factors have been identified, including hypercoagulable states, malignancy, use of oral contraceptives, pregnancy, head injury, infection, and rarely prothrombotic states such as HIT [4]. Very rarely, CVST can be complicated by HIT, leading to worsening of thrombosis and clinical deterioration [3]. HIT is a transient prothrombotic state triggered by exposure to heparin products, predominantly with unfractionated heparin and less likely with low molecular weight heparin (LMWH). The incidence of HIT is approximately 0.5–1% of patients who received therapeutic doses of heparin [3]. In a large analysis by Dhakal et al. where HIT coding was used for the diagnosis of HIT in the National Inpatient Sample (NIS), it was found that 0.065% or 1/1500 of inpatients had HIT [5]. Older age, female gender, higher versus lower doses of heparin, cardiovascular procedures, and UFH versus LMWH are some of the reported risk factors for HIT [6, 7].

The mechanism of HIT is complex. It is an immune-mediated prothrombotic state caused by the formation of antibodies against platelet factor 4 (PF-4) and heparin complex. It then leads to the initiation of a severe procoagulant response by vascular endothelial cells, generating platelets, microparticles, monocytes, and neutrophil extracellular traps, causing thrombocytopenia and thrombosis [8]. Thrombocytopenia is the hallmark of HIT, while thromboembolic complications were found in approximately one third of patients [5].

The most common presentation is a drop in platelet count, occurring at least 4 to 5 days and up to 15 days after exposure to heparin. Rarely, thrombosis can precede the manifestation of thrombocytopenia, making the diagnosis challenging [3]. In patients with CVST and underlying HIT, the most common symptom was headache (60%), followed by altered mental status, visual disturbances, and seizures [9]. HIT should be suspected in patients with a 30–50% decrease in platelet count and recent heparin exposure. In our patient, the workup for HIT was promptly pursued when platelets dropped from 217,000 to 117,000.

The 4T score is the most widely used pretest clinical score for HIT validated by the American Society of Hematology [10]. The score includes thrombocytopenia, the presence of thrombosis, the timing relative to heparin exposure, and other causes of thrombocytopenia. The HIT Expert Probability (HEP) score is another validated pretest clinical test based on the consensus of 26 HIT experts and was found to be equally effective in several studies [11]. In patients with CVST, intracranial hemorrhage (ICH) was the most dreaded complication and was primarily due to occluded cerebral sinus. This highlights the importance of early diagnosis and discontinuation of heparin in cases where HIT is suspected, as it can lead to further worsening of the thrombosis and higher mortality [9].

The diagnosis of HIT is well established. Two types of assays are well described in the guidelines: functional and immunoassays. Immunoassays are easily available screening tests with a high negative predictive value (NPV) but lack specificity, requiring confirmatory testing in positive cases [12]. However, in a large meta-analysis of approximately 15,000 patients, several immunoassays such as polyspecific enzyme-linked immunosorbent assay (ELISA) with an intermediate threshold, polyspecific chemiluminescent immunoassay (CLIA) with a high threshold, lateral flow immunoassay, and immunoglobulin G (IgG)-specific CLIA with a low threshold were found to be associated with sensitivity and specificity of >95% [13]. The serotonin release assay (SRA) is the most widely accepted functional assay for the diagnosis of HIT and detects platelet activation by identifying the serotonin release in the presence of the patient's serum and heparin [14]. It is considered the gold standard test for the diagnosis of HIT with a sensitivity and specificity of >95%.

The first step in the management of HIT with CVST is the discontinuation of all sorts of heparin products, including UFH, LMWH, and heparin flushes. Due to the highly prothrombotic state, anticoagulation with a nonheparin agent is essential [15]. The association of CVST with ICH makes it extremely challenging but requires continued anticoagulation despite the hemorrhage, as discontinuation of the anticoagulation will lead to drastic worsening of thrombosis and hemorrhage [9]. Our patient suffered worsening clinical deterioration despite being on therapeutic dose heparin and showed remarkable clinical improvement after discontinuation of heparin and initiation of argatroban. There are several potential options for anticoagulation in patients with HIT-induced thrombosis, including direct thrombin inhibitor (DTI), fondaparinaux, danaparoid, and direct oral anticoagulants (DOACs) [16]. There is no preferred agent for AC, but argatroban (DTI) is preferred by many experts in the acute setting due to its shorter duration of effect [17].

## 4. Conclusion

HIT is a rare but life-threatening prothrombotic state leading to thrombosis in several distinct locations. CVST is a rare manifestation of HIT requiring prompt discontinuation of heparin and initiation of nonheparin AC to prevent catastrophic consequences. The diagnosis and workup of HIT should be promptly pursued in patients with CVST, thrombocytopenia, and recent exposure to heparin products. In cases where heparin was started for the treatment of CVST and new occurrence of thrombocytopenia, HIT should be strongly suspected. Discontinuation of heparin and initiation of nonheparin AC are the key management steps. Currently, no agent is superior to other, but large prospective studies are needed to better identify the agent of choice.
